# Motor unit properties do not correlate between MUNIX and needle EMG in remote polio in the biceps brachii muscle

**DOI:** 10.1016/j.cnp.2022.12.002

**Published:** 2022-12-23

**Authors:** A. Sandberg

**Affiliations:** Clinical Neurophysiology, Department of Medical Sciences, Uppsala University, Uppsala, Sweden

**Keywords:** MUNIX, MUSIX, Remote Polio, EMG, Macro EMG

## Abstract

**Objective:**

To compare the utility of MUNIX (motor unit number index) with needle EMG in characterizing motor unit (MU) properties in the biceps brachii (BB) muscle in subjects with remote polio.

**Methods:**

Thirty subjects suffering from remote polio were investigated with MUNIX and needle EMG, all with Macro EMG and 16 of these subjects with concentric needle EMG.

**Results:**

Both MUNIX and the needle EMG methods showed abnormal results. Fiber density (FD) was the most sensitive parameter for showing signs of reinnervation. At a group level, the methods showed neurogenic findings, but there was no correlation between the results of the MUNIX and needle EMG investigations.

**Conclusions:**

Both MUNIX and needle EMG are valuable methods for measuring neurogenic involvement in the BB muscle. However, there was a lack of correlation between the MUNIX and needle EMG findings. The cause for this missing correlation may be multifactorial as there are several differences between the methods.

**Significance:**

The reason for the lack of correlation between the MUNIX and needle EMG results is discussed. By combining the needle and surface recorded methods one can obtain more information on the denervation and reinnervation process compared to using just one of the methods alone.

## Highlights

• Both needle EMG and the MUNIX method showed abnormality in the biceps brachii muscle in prior polio, but there was a missing correlation between the needle EMG and the MUNIX parameters.
• Mainly methodological and technical factors may influence the missing correlation between the MUNIX and the needle EMG results.
• By combining the methods greater information about the denervation-reinnervation process is obtained compared to using just one of the methods alone.

## 1 Introduction

Measurement of motor unit (MU) characteristics using electrodiagnostic EMG methods is valuable in the evaluation of neurogenic conditions involving the peripheral motor neuron. In ALS the EMG is used for diagnosis and to follow disease progression (Neuwirth et al., 2017, Troger and Dengler, 2000). The concentric needle EMG (CNEMG) method is preferred in the initial evaluation to establish the diagnosis because it is superior to surface recorded EMG in detecting signs of acute or subacute denervation. When monitoring disease progression, non-invasive surface recorded EMG is preferential to needle EMG methods due to improved patient comfort. In remote polio, EMG is a tool for the diagnosis of status post-polio (Dalakas et al., 1986) and post-polio syndrome (Gawne et al., 1995). Macro EMG results have been discussed as possible markers to predict the risk of new weakness in remote polio (Grimby et al., 1998). Needle EMG methods are rather time consuming and uncomfortable and it may be desirable to use a faster and more comfortable method. The surface recorded Motor Unit Number Index (MUNIX) method has the advantage of a faster non-invasive approach (Nandedkar et al., 2004) and is preferred by most patients over needle EMG. MUNIX measures MU loss and the MUSIX reflects the reinnervation process. It is used in follow up investigations with good reproducibility (Alix et al., 2019, Neuwirth et al., 2016).

In the electrodiagnostic evaluation of neurogenic involvement, information from several muscles increases the diagnostic validity. The proximal arm muscle Biceps Brachii (BB) is a muscle that is often evaluated in remote polio since it is crucial for its weight bearing function in everyday activities. Some investigators have performed MUNIX in the BB muscle, both in ALS (Gawel and Kuzma-Kozakiewicz, 2016) and remote polio (Gawel et al., 2019). In the report regarding ALS, the MUNIX showed no neurogenic involvement in the BB muscle. In the report regarding remote polio a comparison between MUNIX and EMG was performed but the results focused on a sum of results from several muscles together and the separate results from the BB muscle were not reported.

The aim of this report is to evaluate the usefulness of MUNIX in the BB muscle in characterizing MU properties in neurogenic remote polio compared with needle EMG parameters to improve patient comfort.

## 2 Participants

The diagnosis of remote polio had been established clinically by a medical history including an acute phase of weakness with subsequent improvement. A clinical examination by a consultant in rehabilitation medicine or neurology were performed confirming clinical sequelae of paralytic polio (Agre et al., 1995). Needle EMG was used to support the diagnosis. Bilateral muscles were investigated. Due to patient discomfort some investigations were restricted to the one BB muscle that showed predominant involvement clinically. Only the muscles investigated with both Macro EMG and the MUNIX method were included in the study. Exclusion was made if patients had a suspected or confirmed concomitant disorders such as of radiculopathy (3 patients) and diabetic polyneuropathy (1 patient).

Thirty patients with remote polio were included in this study. The patients who were referred from rehabilitation medicine underwent investigation with MUNIX (43 muscles), Macro EMG (43 muscles) and 16 patients underwent CNEMG in 20 muscles. Sixteen women and fourteen men (average age of 65 ± 10 years) were included. The timespan from the acute illness of paralytic polio to the investigation was 46 – 77 years. The EMG investigations were performed by the author, the MUNIX investigations were performed by a technician in addition to the author.

The study was accepted by the local ethics committee (Dnr 2009/301, 2020–04714). All patients gave their informed consent.

## 3 Methods

### 3.1 MUNIX, MUSIX and CMAP

The MUNIX method was originally described in detail by Nandedkar and colleagues in 2004 in an attempt to devise a rapid method to record the status of the innervation of the muscle under study (Nandedkar et al., 2004). Reference values for MUNIX, MUSIX and CMAP were produced in a multicenter study using the original method (Neuwirth et al., 2011).

For this study from the BB muscle in subjects with remote polio, the CMAP active recording silver disc EMG electrode was located over the belly of the BB muscle approximately in the middle of the muscle. The reference electrode was located over the distal part of the BB tendon at the antecubital fossa. Supra-maximal stimulation of the musculocutaneous nerve in the axilla was performed. The recording electrodes were silver disc EMG electrodes (9 mm in diameter).

Recording of the surface electrode interference pattern (SIP) were performed at 9 (minimum 8) different force levels during manual resistance from the examiner using the same placement of the electrodes as used for CMAP recording. The waveforms were analyzed using a program developed and supplied by the inventors.

The absolute and the relative value (the absolute number compared to controls (Neuwirth et al., 2011)) of the parameters were used in the analysis process.

All the MUNIX and needle EMG recordings were made on a commercially available EMG equipment (Keypoint, Medtronic, Copenhagen, Denmark).

### 3.2 Macro EMG

The standard Macro EMG method was applied, developed by Stålberg (Stålberg, 1983). In short, the recording electrode consists of a modified Single Fiber EMG (SFEMG) needle electrode.

The Macro EMG technique has been published in numerical reports, it‘s area of use is mainly for studying the reinnervation process in order to estimate the degree of abnormality (Stalberg, 2011).

The results were expressed as median values of individual amplitudes from at least 15 recorded Macro MUPs. The relative Macro MUP amplitude was expressed as the obtained median value normalized to mean of median values from age matched controls (Stålberg and Fawcett, 1982).

### 3.3 Fiber density

Measurement of FD was performed adjacent to the recording of the Macro MUP using the single fiber surface on the Macro EMG needle. The method is sensitive in detecting signs of reinnervation, e.g. signs of collateral sprouting are revealed as increased FD (Sanders and Stalberg, 1996). Mean relative FD was expressed as the obtained median value normalized to age matched controls publicized by AAEM (Gilchrist, 1992).

### 3.4 Concentric needle EMG

Standard concentric needle EMG (CNEMG) electrodes were used (Ambu, Neuroline Concentric, ref 74038–45/25). Analysis of Motor unit potential (MUP) analysis was performed automatically with Multi MUP analysis (Stalberg et al., 1986) using the Keypoint EMG equipment. Fifteen or more MUPs were required in order to be included in the study.

The results were expressed as mean values of MUP parameters, relative mean values from MUP amplitude and area were normalized for age using the Keypoint EMG equipment specific control values (supplied by Stalbergsoftware.com). Regarding the size index (SI) parameter the results were compared with the values from the study by Bischoff et al (Bischoff et al., 1994).

### 3.5 Force

Force of elbow flexion was assessed manually. The Medical Research Council (MRC) 0–5 scale was used. The force produced could represent contributions from several muscles active during elbow flexion, not only the BB muscle.

### 3.6 Modification of the included material for special analysis

In an attempt to investigate if modification of the included material gave different results the following modifications were performed:

#### 3.6.1 Exclusion of the highest SIP values in the calculation of MUNIX and MUSIX in each muscle.

The 5 highest numbers of SIP were excluded which left the 4 lowest SIP values that build up the regression line in the MUNIX analysis. This was performed in order to investigate whether the exclusion of the MUs with the highest threshold for activation have any impact on the relationship between the MUNIX/MUSIX and the needle EMG parameters.

#### 3.6.2 Exclusion of data where a situation of possible fragmentation of the MU or failure of reinnervation may be present.

**Fig. 1:**
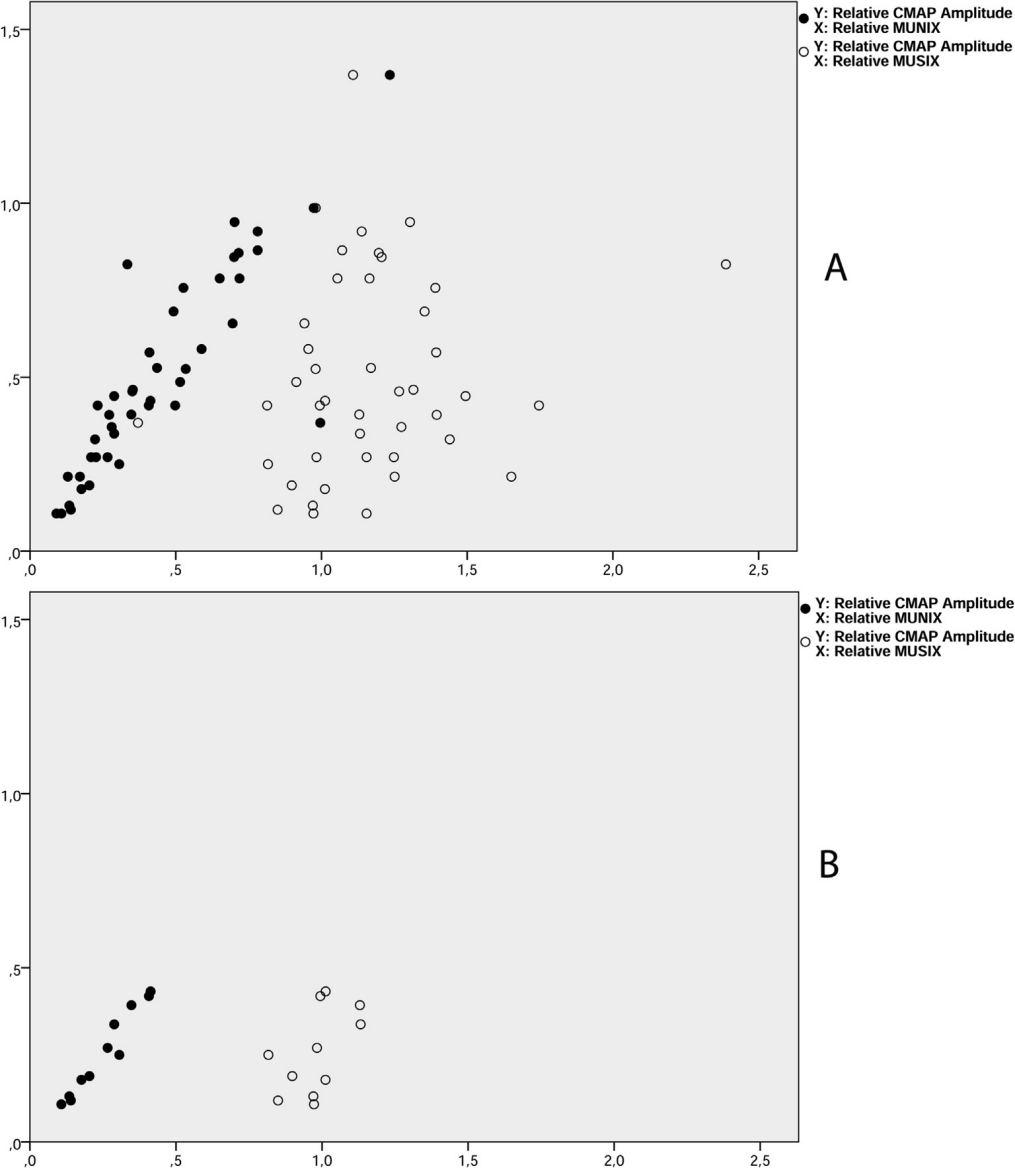
A) The relationship between the relative CMAP on the x-axis and the relative MUNIX (filled symbols) and MUSIX (unfilled symbols) showed on the y-axis. There was a significant correlation between the relative CMAP and the relative MUNIX. There was no correlation between the relative CMAP and relative MUSIX. B) CMAP, MUNIX and MUSIX from the 11 muscles, showing the combination of decreased CMAP, decreased MUNIX and non-elevated MUNIX which is the combination expected in fragmentation or failure of reinnervation of the denervated MUs. Data from these 11 muscles is excluded in the “modification of the included material for special analysis”, regarding the effect from fragmentation and failure of reinnervation on the data.

Some recordings show the expected findings of low CMAP, low MUNIX and no elevation of the MUSIX when a possible fragmentation or insufficient reinnervation of the MUs was present (Fig. 1B). A modified analysis excluding data from the muscles with the above data combination was performed to investigate if there was a correlation between MUNIX and needle EMG parameters when possible fragmentation or insufficient reinnervation was excluded. 32 muscles regarding MUNIX and Macro data were included in the analysis, 15 regarding MUNIX and CNEMG.

#### 3.6.3 Exclusion of muscles with normal Macro amplitude.

The relationship between the MUNIX/MUSIX and needle EMG parameters in muscles that show increased macro amplitude as a sign of reinnervation after denervation was investigated, muscles without signs of reinnervation were excluded. This modified analysis only including cases with neurogenic involvement was performed to investigate whether exclusion of normal cases increased the correlation between the MUNIX and needle EMG methods. With this setup there were 27 muscles analyzed with regards to the MUNIX/MUSIX and Macro amplitude including FD. Regarding the CNEMG parameters 12 muscles were included in this modified analysis.

#### 3.6.4 Exclusion of muscles which showed some degree of complex CMAP source.

Some of the recordings showed complex or unusually shaped CMAP responses due to CMAP “contamination” from other muscles or short latency take off due to distal stimulation of the musculocutaneous nerve. This optimization of the CMAP were performed in an attempt to investigate the effect on the relationship with the needle EMG parameters. With this setup there were 30 muscles analyzed regarding the MUNIX/MUSIX and Macro amplitude including FD. Regarding the MUNIX/MUSIX and CNEMG parameters, data from 15 muscles were included in the analysis.

### 3.7 Statistical analysis

The Pearson correlation coefficient in bivariate correlations was used. When interval scale was not present Spearman correlation was used. All statistics were performed with the commercially available software SPSS. Differences were considered significant at P < 0,05.

## 4 Results

### 4.1 Baseline results

The baseline results for the MUNIX, MUSIX, needle EMG parameters and force measured using the MRC scale are shown in Table 1.

**Table 1:** The number and percentage of abnormal surface and needle parameters. The relative mean value for the different parameters are also shown and the resulting calculated percentage deviation from the control values are summarized.

	n abn/n normal	% abn	Mean	Range	Mean rel value	Range relative value	% deviation from control value
MUNIX (n = 43 Muscles)	26/17	60	80	16–240	0,44	0,09–1,2	130
MUSIX (n = 43 Muscles)	21/22	49	51 uV	16–103 uV	1,2	0,37–2,4	16
CMAP (n = 43 Muscles)	22/21	51	3,9 mV	0,80–12 mV	0,50	0,11–1,4	100
Macro MUP Amplitude (n = 43 Muscles)	27/16	63	320 uV	65–1180 uV	2,9	0,53–11	190
FD (n = 43 Muscles)	41/2	95	2,3	1,5–4,2	1,6	1,1–2,7	63
CNEMG MUP Amplitude (n = 20 Muscles)	8/12	40	770 uV	350–2800 uV	1,6	0,75–6,0	63
CNEMG MUP Area (n = 20 Muscles)	12/8	60	1400 uVms	400–6200 uVms	2,3	0,65–10	130
CNEMG SI (n = 20 Muscles)	5/15	25	1.03	0,19–3,1	1,6	0,3–4,7	59
Force MRC (n = 38 Muscle groups)	3/35	8	–	MRC 4–5	–	–	–

The FD parameter was the most sensitive parameter for detection of abnormality. The Macro mean MUP, CNEMG area and MUNIX parameters shows the greatest degree of abnormality. The MUSIX showed least degree of abnormality. Also elbow flexion force (MRC) is shown in which just 8 % of the tested muscle groups showed reduced force.

### 4.2 Correlations of results

The correlations between the relative parameters from all methods are shown in Table 2.

**Table 2:** The correlation between the relative parameters. Note the non-significant (NS) correlation between the MUNIX and the needle EMG parameters.

Correlation, relative values	CMAP	MUNIX	MUSIX	CNEMG MUP ampl	CNEMG MUP area	CNEMG SI	Macro MUP ampl	FD	Force
CMAP		0,86 (p < 0,001)	NS	NS	NS	NS	NS	NS	NS
MUNIX	0,86 (p < 0,001)		NS	NS	NS	NS	NS	NS	NS
MUSIX	NS	NS		NS	NS	NS	NS	NS	NS
CNEMG MUP ampl	NS	NS	NS		0,95 (p < 0,001)	0,91 (p < 0,001)	0,79 (p < 0,001)	0,79 (p < 0,001)	NS
CNEMG MUP area	NS	NS	NS	0,95 (p < 0,001)		0,92 (p < 0,001)	0,66 (p < 0,01)	0,65 (p < 0,01)	NS
CNEMG SI	NS	NS	NS	0,91 (p < 0,001)	0,95 (p < 0,001)		0,58 (p < 0,001)	0,71 (p < 0,001)	NS
Macro MUP ampl	NS	NS	NS	0,79 (p < 0,001)	0,66 (p < 0,01)	0,76 (p < 0,001)		0,78 (p < 0,001)	NS
FD	NS	NS	NS	0,79 (p < 0,001)	0,65 (p < 0,01)	0,71 (p < 0,001)	0,78 (p < 0,001)		NS
Force	NS	NS	NS	NS	NS	NS	NS)	NS	

**Fig. 2:**
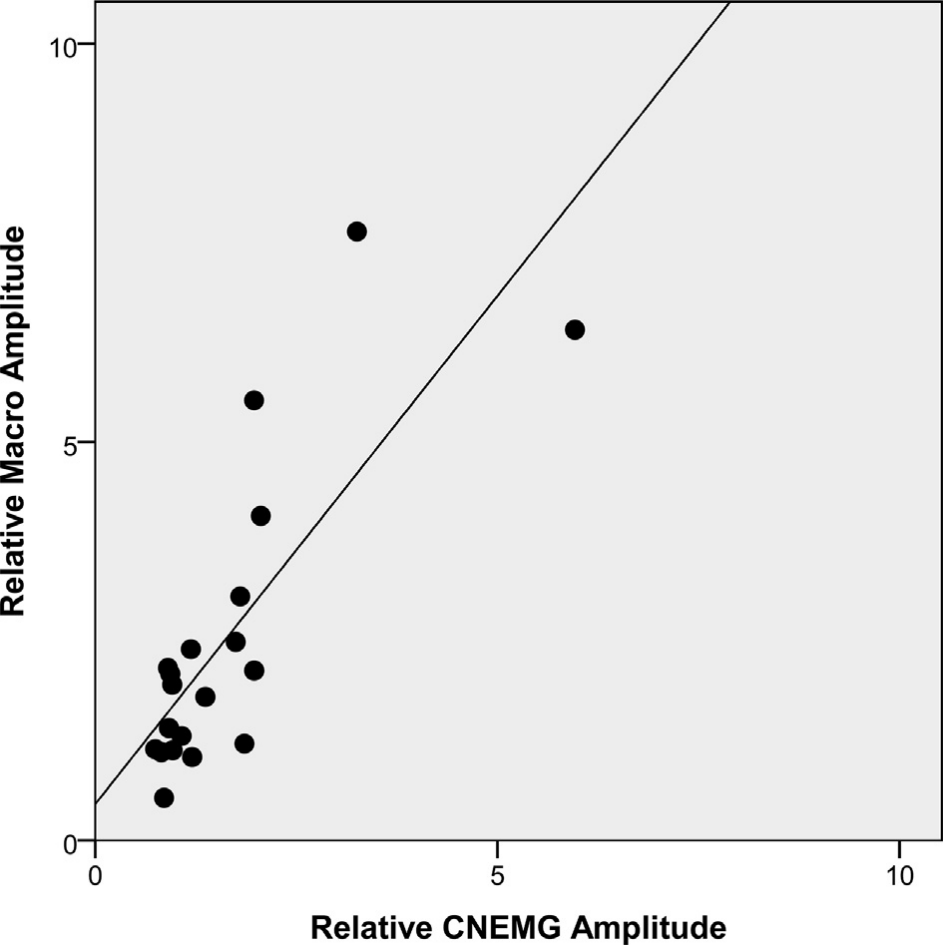
The significant relationship between the relative Macro MUP amplitude and the relative CNEMG MUP amplitude.

**Fig. 3:**
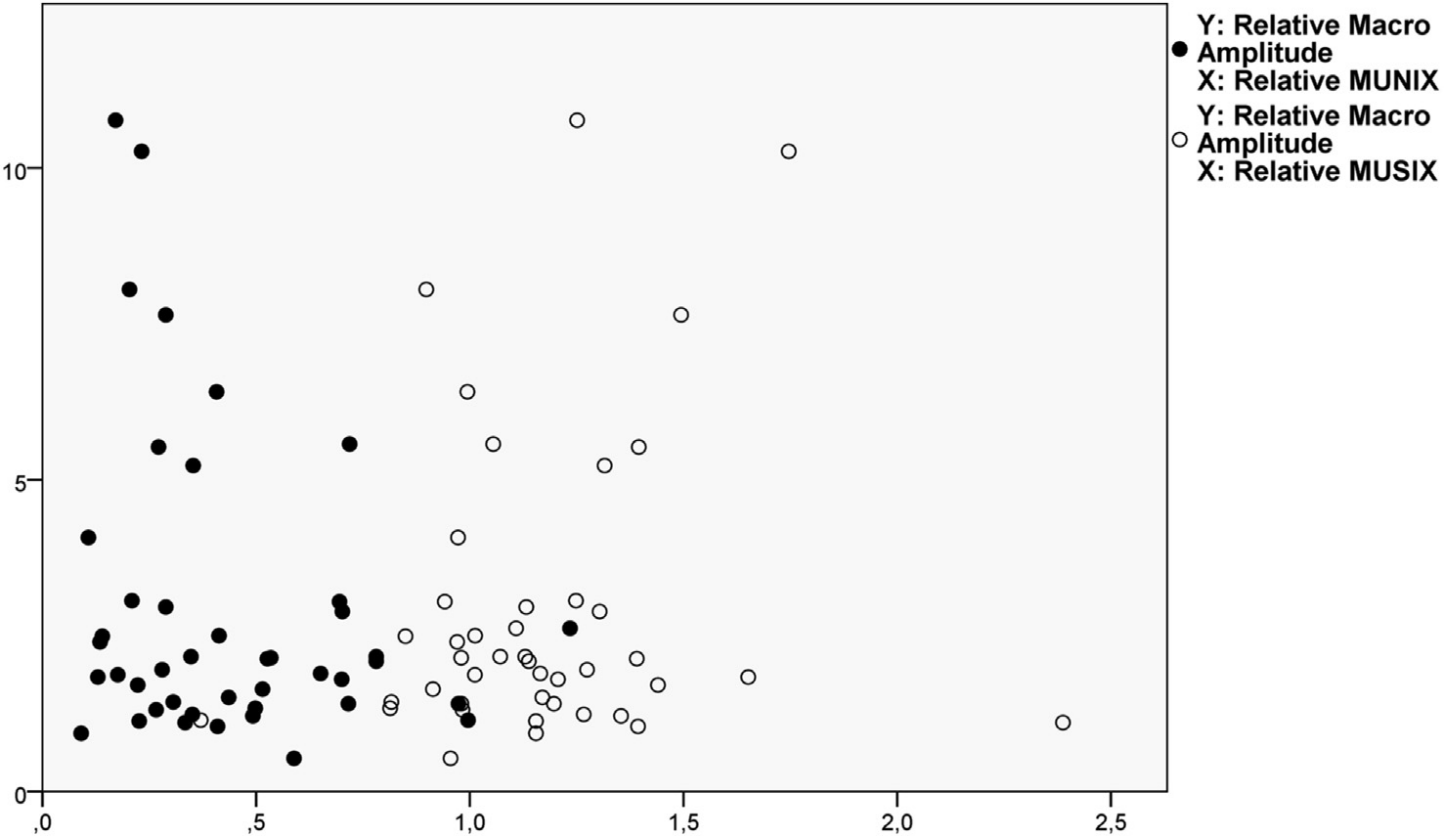
The lack of correlation between the relative Macro MUP amplitude (on the x-axis) and the MUNIX (filled symbols) and MUSIX unfilled symbols) showed on the y-axis.

**Fig. 4:**
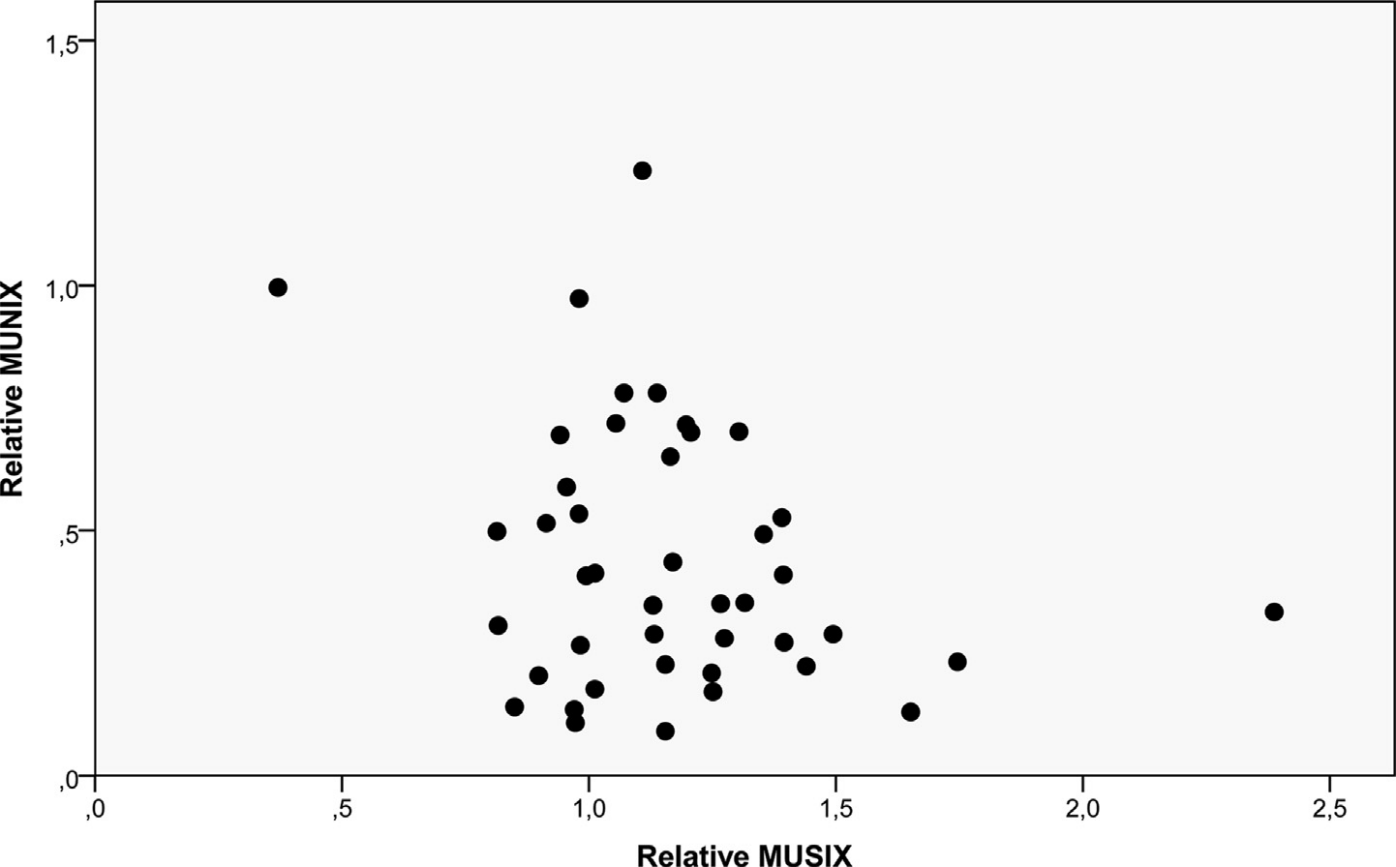
The lack of correlation between the relative MUNIX on the y-axis and the relative MUSIX on the x-axis.

There was a positive correlation between the relative CMAP and relative MUNIX, Fig. 1A. There were also positive correlations between the needle EMG parameters, some of which are shown in Fig. 2. There was no correlation between the CMAP and the MUSIX, Fig. 1A. There was no correlation between the MUNIX/MUSIX and the different needle EMG parameters, Fig. 3. The MUNIX showed no correlation with MUSIX, Fig. 4. The force showed no correlation with the MUNIX or the needle EMG parameters.

### 4.3 Results following modifications

#### 4.3.1 Highest SIP values excluded.

Exclusion of the highest SIP data from every investigation gave a significantly negative correlation between the relative MUNIX and MUSIX (-0,41, p < 0,01). There was however no correlation between the relative MUNIX/MUSIX and the relative needle EMG parameters. This exclusion of the MUNIX and MUSIX analysis of the MUs with the high threshold did not increase the correlation with the needle EMG parameters.

#### 4.3.2 Fragmentation of the MUs or failure of reinnervation excluded.

There was a significant correlation between the MUNIX and the SI (-0,55, p = 0,035). Also the MUSIX showed a correlation with SI (0,52, p = 0,049). Otherwise no correlation was found between the MUNIX and the needle EMG parameters.

The MUNIX AND MUSIX also showed significant correlation (-0,52, p < 0,01).

#### 4.3.3 Normal Macro EMG excluded.

There was no correlation between the relative MUNIX/MUSIX and the relative needle EMG parameters when analyzing only muscles with signs of reinnervation measured as increased Macro amplitude. The mean relative Macro amplitude was 3,9 in this group when the muscles with normal Macro MUP amplitude was excluded.

#### 4.3.4 Complex CMAP source excluded.

There was no significant correlation between the relative MUNIX/MUSIX and the relative EMG parameters. However, there was a borderline relationship between the relative MUSIX and the relative Macro amplitude (0,353, p = 0,056).

## 5 Discussion

In this report on motor unit properties in the biceps brachii muscle in subjects with remote polio there were signs of MU loss and reinnervation indicated with MUNIX and the needle EMG methods. The FD was the most sensitive parameter for detecting abnormality, with abnormal values recorded in 95 % of the muscles. The CNEMG MUP amplitude and SI showed least sensitivity.

The different methods showed different degree of abnormality. MUNIX and Macro amplitude showed largest degree of abnormality, MUSIX showed the lowest degree of abnormality.

Although the different methods showed signs of motor neuron involvement, no clear association was found between the MUNIX/MUSIX parameters compared to the needle EMG parameters. This missing relationship between the methods was not the case in a previous paper comparing MUNIX and Macro EMG in the large tibial anterior muscle (Sandberg et al., 2011). Possible reasons for the lack of correlation between the results of the different methods are discussed below.

### 5.1 Physiological or anatomical factors which may influence the measurement of the MU properties with the different methods is hereby discussed.

#### 5.1.1 Different MU populations recorded with the different methods due to physiological properties of the MUs

One reason for the missing correlation may be due to the different populations of MUs measured with the two techniques. MUNIX and MUSIX are measured from both low and high threshold MUs while the needle EMG signals are measured from low threshold MUs only. To test this hypothesis regarding different results depending on different MU populations an experimental set-up in the analysis of MUNIX and MUSIX were performed in order to exclude the contribution from the surface interference pattern at high activation. Practically approximately the highest half of the SIP measurement points were excluded, there was still no relationship between the EMG and the MUNIX/MUSIX parameters. Nevertheless, it is unknown to me if the result from the reduced dataset of SIP just represents a technical error in the calculation of MUNIX or drop out of the data from the high threshold MUs. Therefore it is still possible that the different MU populations analyzed with the different methods may be a reason for the missing correlation between the MUNIX/MUSIX and EMG results, as was the situation in a report regarding missing correlation between CNEMG and MUNIX regarding a small hand muscle in ALS (Jacobsen et al., 2018).

#### 5.1.2 Different MU populations detected with MUNIX versus EMG due to anatomical properties

Another reason for the missing correlation between the MUNIX and EMG parameters may be the difference in MU populations detected with MUNIX versus EMG due to anatomical properties of the recorded MUs. Different MU sizes at different depths within the vastus lateralis muscle have been reported (Knight and Kamen, 2005). Whether this is also the case in the BB muscle is not known. However, there is a report of differences in the MU behavior during relaxation in different parts of the BB muscle (Denier van der Gon et al., 1985) which indicate regional differences regarding MU properties. Since the MUNIX method is recorded with surface electrodes the main MU contribution under recording is from superficially located MUs while the contribution from deeply located MUs is suppressed (Barkhaus and Nandedkar, 1994). Since needle EMG signals are recorded both from superficial and deep MUs, this may have implications if anatomical differences in the MU size is present. The BB muscle consists of the long and short heads. It is possible that the two heads have different MU properties or different neurogenic involvement and if the EMG is recorded preferably from one of the heads and the MUNIX is recorded preferably from the other head one may record different MU populations. Recording from different locations in the BB muscle with the two main methods may be a reason for the missing correlation between the MUNIX and EMG.

#### 5.1.3 Failure of reinnervation or fragmentation of the MUs

Another reason for the non-concordance of the results of the MUNIX and the EMG methods could be the possibility of failure of reinnervation or fragmentation of the MUs (Dalakas et al., 1986, Wiechers and Hubbell, 1981). If a significant amount of muscle fibers remains orphaned after denervation due to failure of the reinnervation process or the MU gets fragmentated due to AHC deficiency (Borg et al., 1988) this may occur. If this affects for instance the largest MUs (with the greatest degree of reinnervation which may be the case in remote polio (Grimby et al., 1998)) and the MU recruitment order size principle by Henneman is preserved (as expected in motor neuron disorders (Milner-Brown et al., 1974)), Macro EMG misses this effect on the largest MUs while the MUNIX parameters and CMAP take this effect into account. In this material 11 muscles showed results which is expected when fragmentation or failure of reinnervation after denervation is present (Fig. 1B). The resulting significant relationship between the MUNIX parameters and the SI shows that the situation of defective reinnervation or fragmentation of the MU at least to some extent explains the lack of relationship between the results of these methods. Also, the lack of correlation between the MUNIX and MUSIX results may also be explained by this situation.

### 5.2 Technical factors which may have an influence on the result of the MU properties measured with the different methods is hereby discussed.

#### 5.2.1 Differences in recording properties between the electrode types regarding electrode location, electrode size and EMG signal frequency content

The difference in the proximity of the recording electrodes to the generators of the MU potentials is known to be essential for the properties of the recorded MUPs (Barkhaus and Nandedkar, 1994, King et al., 1997, Stalberg and Fawcett, 1982) and can have an impact on the results. The skin surface recorded MUPs are dependent on the distance between the MU and the skin surface, including the thickness of the subcutaneous fat layer (Barkhaus and Nandedkar, 1994). The MUP amplitude decreases with increasing distance between the active MU and the active electrode (Barkhaus et al., 2006). Equally large MUs recorded deep in the muscle shows lower MU amplitude and area compared to more surface located MUs (Barkhaus et al., 2006, Roeleveld et al., 1997). Needle EMG recorded MUPs do not depend on the recording depth in the muscle in the same way as surface recorded MUPs do, thus resulting in less variability of the needle recorded MUs.

The MUNIX result is dependent on the proximity to the innervation zone (Gao et al., 2020). Macro EMG commonly doesńt depend on the recording position in the MU, but a 50 % reduction of the MUP amplitude has been reported (Stalberg and Fawcett, 1982) when recording is performed in the periphery of the MU.

Different size of surface electrodes show different values of CMAP and surface recorded MUPs resulting in different MU counts for the different sized electrodes (Barkhaus et al., 2006). The measurements from needle electrodes with smaller recording areas used in CNEMG and FD recordings dońt suffer from the shunting effect as much, compared with bigger electrodes such as Macro and larger surface electrodes. This factor could have an implication on the missing correlation between the surface and needle recorded MUPs.

It is shown that the frequency content of the recorded MUPs is dependent on the distance from the muscle fiber to the recording site with small electrodes (Gath and Stalberg, 1977). The conductivity is not per se the cause for the relative suppression of higher frequency for remotely located MUs (Gabriel et al., 1996). Instead, in the case of Macro EMG versus CNEMG the low frequency content from distant MUs is not as suppressed in the Macro as in the CNEMG (Stålberg, 1983). The location of the surface EMG electrodes over the skin may also cause the measurements to suffer from suppression of the higher frequency content from the filtering effect from the subcutis (Petrofsky, 2008).

EMG signals with higher frequency content compared to EMG signals with lower frequency content from densely collaterally reinnervated MUs may be at a greater risk of the effects of phase cancellation which results in lower amplitude EMG signals than expected for the MUs with the higher frequency content (Nandedkar et al., 1988, Sandberg et al., 1999). This may be a reason for the missing correlation between CNEMG and MUNIX since MUNIX may depend on the filtering effect from the subcutis.

#### 5.2.2 MUNIX and MUSIX have low sensitivity for changes in MU amplitude

Another factor that may impact the results that has been discussed recently is the possibility that the MUNIX and the MUSIX have low sensitivity for changes in MU amplitude. In a simulation study (Li et al., 2012) the effect on the MUNIX and MUSIX was reported to be less than expected in simulated change of the SIP in comparison to what this change in MU amplitude has on other MUNE methods. Another study (Bostock et al., 2019) showed when motor unit potentials overlap extensively, information about motor unit size and number was lost and MUNIX depends only on CMAP parameters. This effect was reported to have the greatest influence on normal or small MUs. In an attempt to check if this is true for this material which has a rather low degree of abnormality, a statistical analysis was performed only including the subset of muscles with abnormal Macro amplitude values which excludes the Macro recordings with the lowest MU amplitude. Still, there were no relationship between the MUNIX/MUSIX and the EMG parameters. The apparent relationship between CMAP and the MUNIX and the influence on the MUNIX from the CMAP can however not be ignored as a contributing reason for the lack of relationship between MUNIX and the needle EMG methods.

#### 5.2.3 Different control values

Different control values used for the different methods as a cause for the missing relationship may be of importance. In this study control values for the CMAP, MUNIX, MUSIX, Macro amplitude, FD and SI are used which have been published in peer-review reports (Gilchrist et al., 1992, Bischoff et al., 1994, Neuwirth et al., 2011, Stalberg and Fawcett, 1982). For the MUP amplitude and MUP area the EMG equipment specific control values are used. Since there is no clear systemic relationship between the MUNIX and the EMG-parameters this may not be the cause.

#### 5.2.4 CMAP issues

Technical reasons such as wrong CMAP sources from other muscles and sub-optimization of the active electrode may interfere with the MUNIX results. There are reports indicating a noticeable positive relationship between the CMAP and the MUNIX (Bostock et al., 2019), this is also the case in this study. CMAP contribution from additional or wrong muscles has been reported (Neuwirth et al., 2018) to have an impact of the MUNIX result but not on the MUSIX which is less dependent on the CMAP (Li et al., 2012). To investigate the impact of possible CMAP sources from other muscles than BB an exclusion of the cases with complex CMAP source was made resulting in a borderline but insignificant correlation between the relative macro amplitude and the relative MUSIX. There was no such tendency to a borderline correlation between the EMG parameters and MUNIX. Wrong CMAP source could therefore not be totally excluded as a possible cause of the lack of correlation between at least the MUSIX and the EMG parameters.

## 6 Conclusion

To conclude, the MUNIX including the MUSIX and the needle EMG reflect the denervation and reinnervation process in the BB muscle. MUNIX shows the degree of loss of MUs while MUSIX and needle EMG MUP analysis measure the degree of reinnervation, as does the FD which is the most sensitive parameter for the detection of reinnervation. MUNIX and Macro EMG MUP amplitude showed the largest degree of abnormality while MUSIX showed more modest signs of reinnervation. While both the needle and the surface recorded methods show neurogenic involvement on group level, there was no correlation between the MUNIX and the needle EMG parameters on the individual muscle level. This lack of correlation between the methods is different to the result in an earlier report regarding MUNIX and Macro EMG in the tibial anterior muscle. The cause for this missing relationship may be multifactorial since there are differences between the methods. There are several issues that should be considered which can affect the result when performing needle EMG and MUNIX in the BB muscle, namely:

- Different MU populations are recorded with the different methods, depending on recruitment order and anatomical properties of the MUs;
- Failure of reinnervation after denervation or fragmentation of the MUs give different results from the methods;
- Differences in recording properties of the EMG signals between the electrode types regarding electrode location, electrode size and phase cancellation phenomenon;
- Methodological issues regarding MUNIX calculation may be present when smaller MU size is analyzed;
- CMAP contribution from additional or wrong muscles may interfere with the results.
- In combining the methods greater information about the denervation-reinnervation process is received compared with using just one of the methods alone.

## Disclosure

The author has no potential conflicts of interest to be disclosed.

## Declaration of Competing Interest

The author declare that he have no known competing financial interests or personal relationships that could have appeared to influence the work reported in this paper.
